# RETRACTED: Mustafa et al. Exogenous Application of Green Titanium Dioxide Nanoparticles (TiO_2_ NPs) to Improve the Germination, Physiochemical, and Yield Parameters of Wheat Plants Under Salinity Stress. *Molecules* 2022, *27*, 4884

**DOI:** 10.3390/molecules31091538

**Published:** 2026-05-06

**Authors:** Nilofar Mustafa, Naveed Iqbal Raja, Noshin Ilyas, Fozia Abasi, Muhammad Sheeraz Ahmad, Maria Ehsan, Asma Mehak, Imran Badshah, Jarosław Proćków

**Affiliations:** 1 Department of Botany, PMAS Arid Agriculture University, Rawalpindi 46300, Punjab, Pakistan; drnaveedraja@gmail.com (N.I.R.); noshinilyas@uaar.edu.pk (N.I.); abasifozia@gmail.com (F.A.); 14-arid-4355@student.uaar.edu.pk (M.E.); amee.92@hotmail.com (A.M.); imranbadshah686@gmail.com (I.B.); 2 Department of Biochemistry, PMAS Arid Agriculture University, Rawalpindi 46300, Punjab, Pakistan; dr.sheeraz@uaar.edu.pk; 3 Department of Plant Biology, Institute of Environmental Biology, Wrocław University of Environmental and Life Sciences, ul. Kożuchowska 5b, PL-51-631 Wrocław, Poland

The Journal retracts the article “Exogenous Application of Green Titanium Dioxide Nanoparticles (TiO_2_ NPs) to Improve the Germination, Physiochemical, and Yield Parameters of Wheat Plants Under Salinity Stress” [1] cited above.

Following publication, concerns were brought to the attention of the Editorial Office regarding discrepancies between data presentation and description, the use of unusual phrases, and the origins of Figure 1 presented in this article [1].

In accordance with the journal’s standard editorial procedures, an investigation was conducted by the Editorial Office and Editorial Board that confirmed methodological shortcomings along with other scientific flaws that seriously impact the reproducibility and validity of this study. Additionally, the presence of unusual phrases and other image-related concerns could not be fully resolved. Consequently, the Editorial Board has lost confidence in the reliability of the findings and decided to retract this publication [1], as per MDPI’s retraction policy (https://www.mdpi.com/ethics#_bookmark30).

This retraction was approved by the Editor-in-Chief of the journal *Molecules.*

The authors have been informed of this retraction and have indicated their disagreement with this decision.
